# Early and late-onset preeclampsia: effects of DDAH2 polymorphisms on ADMA levels and association with DDAH2 haplotypes

**DOI:** 10.61622/rbgo/2024AO19

**Published:** 2024-03-15

**Authors:** Fernanda Santos Mendes, Marcelo Rizzatti Luizon, Ana Cristina dos Santos Lopes, Daniela Alves Pereira, Fernanda Cristina Gontijo Evangelista, Lara Carvalho Godoi, Luci Maria Dusse, Patrícia Nessralla Alpoim

**Affiliations:** 1 Universidade Federal de Minas Gerais Belo Horizonte MG Brazil Universidade Federal de Minas Gerais, Belo Horizonte, MG, Brazil.

**Keywords:** Pre-eclampsia, Asymmetric dimethylarginine, Dimethylarginine dimethylaminohydrolase 2 gene, Nitric Oxide Synthase Type III/ genetics, Nitric Oxide Synthase, Haplotypes, Polymorphism, genetic, Pregnant women, Genotype

## Abstract

**Objective:**

To examine whether the *DDAH2* promoter polymorphisms -1415G/A (rs2272592), -1151A/C (rs805304) and -449G/C (rs805305), and their haplotypes, are associated with PE compared with normotensive pregnant women, and whether they affect ADMA levels in these groups.

**Methods:**

A total of 208 pregnant women were included in the study and classified as early-onset (N=57) or late-onset PE (N =49), and as normotensive pregnant women (N = 102).

**Results:**

Pregnant with early-onset PE carrying the GC and GG genotypes for the *DDAH2* -449G/C polymorphism had increased ADMA levels (P=0.01). No association of *DDAH2* polymorphisms with PE in single-locus analysis was found. However, the G-C-G haplotype was associated with the risk for late-onset PE.

**Conclusion:**

It is suggested that *DDAH2* polymorphisms could affect ADMA levels in PE, and that *DDAH2* haplotypes may affect the risk for PE.

## Introduction

Preeclampsia (PE) is characterized by hypertension after 20 weeks of gestation and other clinical criteria, which may include proteinuria.^(1)^ PE is a multisystem disorder that targets the kidneys, liver, endothelium, brain, and coagulation processes, thereby leading to complications such as acute renal failure, hepatic rupture and cerebrovascular disease. Moreover, fetal complications can be observed in preeclamptic women, such as intrauterine growth restriction and preterm delivery.^(2)^ Therefore, PE is a major cause of maternal and/or perinatal morbidity and mortality.^(2,3)^ The pathogenesis of PE is related to placental disorders in early pregnancy, which is followed by the onset of generalized inflammation and progressive endothelial lesion. PE was proposed to be classified according to the onset of symptoms into early-onset (<34 weeks of gestation) and late-onset PE (≥34 weeks of gestation).^(4)^

Nitric oxide (NO) is synthesized from L-arginine by the NO synthases (NOS), and it acts as a potent vasodilator and an important regulator of vascular resistance.^(5)^ Diminished NO availability has been shown to be associated with pathophysiological mechanisms of PE.^(6)^ In this context, polymorphisms of the endothelial NOS (*NOS3*) gene were shown to affect nitrite concentrations (a marker of endogenous NO formation) in healthy subjects.^(7)^ Moreover, *NOS3* polymorphisms were found to be associated with essential hypertension^(8)^ and with hypertensive disorders in pregnancy,^(9)^ including with early and late severe PE.^(10)^ However, other parameters may affect NO levels beyond the genetic polymorphisms of *NOS3*.

Asymmetric dimethylarginine (ADMA), an endogenous inhibitor of NO synthesis, competes with L-arginine producing citrulline and dimethylamine. ADMA is formed by post-translational methylation, involving the addition of methyl groups to arginine residues by methyltransferases.^(11)^ ADMA is released into the cytosol when these proteins with arginine residues are hydrolyzed, thereby being an obligatory product of protein turnover.^(11,12)^ Our group and others have reported higher ADMA levels in preeclampsia,^(13,14)^ and that circulating ADMA is elevated before the development of PE.^(15)^ Although the kidney plays an important role in the elimination of ADMA, its conversion by dimethylarginine dimethylaminohydrolase (DDAH 1 and 2) into citrulline and dimethylamine is the most important metabolic pathway.^(11)^

*DDAH2* is highly expressed in the placenta, whereas the protein turnover is increased in the developing fetoplacental unit.^(16)^ These findings suggest an important role of *DDAH2* in regulating the function of trophoblasts by their effect on NO production.^(17)^ Although the relationship among DDAH, increased ADMA levels and the impaired NO synthesis is already identified, the regulation of gene expression and DDAH activity remains unknown.^(16,18)^ In addition, no previous study has examined whether polymorphisms and haplotypes of *DDAH2* gene affect ADMA levels or are associated with PE, including early-onset and late-onset PE.

In the present study, we examined whether the *DDAH2* polymorphisms -1415 G/A (rs2272592), -1151A/C (rs805304) and -449G/C (rs805305), and the haplotypes formed by them, are associated with early-onset or late-onset PE. Moreover, we examined whether genotypes for the *DDAH2* polymorphisms affect ADMA levels in normotensive pregnant women and in early-onset or late-onset PE.

## Methods

This study was approved by the Ethics Committee of the Federal University of Minas Gerais, Brazil (ETIC 0530.0.203.000-09). Informed consent was obtained from all participants. This research was carried out according to the Declaration of Helsinki, as revised in 2008, without interference with medical recommendations or prescription.

This case-control study included 208 Brazilian pregnant women in the third trimester of gestation distributed in three groups: normotensive pregnant controls (NP, N = 102), early-onset PE (N = 57) and late-onset PE (N = 49). Preeclamptic women were selected at the time of hospital admission, while normotensive pregnant women were selected at a routine clinic visit, when the blood samples were collected.

The inclusion criteria for PE was defined by systolic blood pressure ≥ 160 mmHg or diastolic blood pressure ≥ 110 mmHg, at least in two occasions and four hours apart, after 20 weeks of gestation. Other clinical signs and symptoms were also considered, including cerebral or visual disturbances, progressive renal insufficiency and impaired.^(3)^ Pregnant women with PE were grouped according to the gestational age on the onset of symptoms as early-onset (gestational age <34 weeks) and late-onset (gestational age ≥34 weeks).^(4)^ The normotensive pregnant group included healthy women with no history of PE or hypertension.

The common exclusion criteria for all groups were: chronic hypertension, diabetes mellitus, cancer, coagulation disorders, cardiovascular, autoimmune, hepatic, renal and inflammatory/infectious diseases. Clinical and laboratory data were obtained from medical records and interviews with the participants.

Maternal ADMA plasma levels were evaluated by ELISA (Diagnostika GmbH, Koln, Germany), as previously described.^(13)^

DNA was extracted and purified from whole blood, previously collected in EDTA, using Biopur Mini Spin Kit® (Biometrix, Brazil), according to the manufacturer´s instructions. The DNA samples were stored at -80°C until the analysis.

The -1415G/A polymorphism (rs2272592) was assessed by real time polymerase chain reaction (RT-PCR) using TaqMan SNP Genotyping Assay C___3233670_1, in StepOne Real-Time PCR equipment System (Thermo Scientific®, Massachusetts, EUA). In all PCR reactions, samples from previously genotyped subjects were included as controls. In addition, 10% of samples were re-genotyped in order to confirm the results.

The -1151A/C (rs805304) and -449G/C (rs805305) polymorphisms were genotyped by PCR followed by restriction fragment length polymorphism (PCR-RFLP) analysis, using the following primers: 5´-CCGTTGCGCTGTTCTGAGGTCTAC-3´ (sense), and 5´-CGGCTCCGGGGCATTGTCTA-3´ (antisense) for the -1151A/C polymorphism; 5´-CCTTCTCGTTCGGGTATTCAG-3´ (sense), and 5´-TCCAGACCTTCCGCTCCT-3´ (antisense) for the -449G/C polymorphism.

The final volume of PCR reaction mixture was 20 μL and contained 1 mM of each primer (Invitrogen®, São Paulo, Brazil), 0.2 mM deoxynucleoside triphosphate (Ludwig Biotec®, Alvorada, Brazil), 2.5 μL of 10X PCR Buffer and 0.5 units Taq polymerase (Thermo Scientific®, Massachusetts, EUA). The reactions were performed in a 2720 PCR thermocycler (Applied Biosystems®, California, USA) with initial denaturation step at 94°C for 5 min, followed by 35 cycles consisting of denaturation at 94°C for 40 seconds, 50 seconds at 71°C and 56°C for primer annealing of the -1151A/C and -449G/C polymorphisms, respectively, and extension at 72ºC for 30 seconds.

The PCR products of -1151A/C and -449G/C were submitted to endonuclease digestion for 4 h at 37°C with the enzyme *Mnl*1 (Thermo Scientific®, Massachusetts, EUA) and for 3.5 h at 30°C with the enzyme *Sma*1 (Thermo Scientific®, Massachusetts, EUA), respectively, in a single reaction. Fragments were visualized on 8% polyacrylamide gel electrophoresis stained by silver nitrate.

Haplotype frequencies were estimated by using Haplo.stats package version 1.4.4 (http://cran.r-project.org/web/packages/haplo.stats/index.html), which computes maximum likelihood estimates of haplotypes and differences in haplotype frequencies using haplotype-specific scores to test for association.^(19)^ The possible haplotypes including the alleles for the *DDAH2* polymorphisms -1415G/A (rs2272592), -1151A/C (rs805304) and -449G/C (rs805305) were: G-C-C, G-A-G, A-A-G, G-C-G and G-A-C. The odds ratio and 95% confidence intervals were calculated for each haplotype. Only the haplotypes with frequencies >5% were taken into consideration in the analysis. A value of P=0.01 (0.05/5, the number of haplotypes) was considered significant to correct for the number of comparisons made (Bonferroni’s correction). Non-additive effects were selected to score haplotypes, and we have also considered dominant and recessive models.

Statistical analyses were performed with SPSS software (SPSS: An IBM Company, version 17.0, New York, USA) and STAT pages. Data normality was tested by Shapiro–Wilk test. Nonparametric variables (age, gestational age, body mass index, weight gain, systolic and diastolic blood pressures) were compared by the Mann-Whitney test. Categorical variables (*DDH2* polymorphisms) were compared between groups by Pearson Chi-Square test. The distribution of genotypes for each polymorphism was assessed for deviation from the Hardy–Weinberg equilibrium, and differences in genotype and allele frequencies between groups were assessed using χ^2^ tests. A value of P <0.05 was considered statistically significant.

## Results

The clinical and demographic characteristics of the participants enrolled in this study are shown in table 1. There were no significant differences when comparing age and body mass index (BMI) among the normotensive and early-onset and late-onset PE groups (P>0.05) (Table 1). Weight gain during pregnancy was higher in women with late-onset PE when compared to normotensive pregnant controls (p<0.001). As expected, systolic and diastolic blood pressure were significantly higher in early-onset and late-onset PE groups compared to normotensive pregnant women (P<0.001), while gestational age differed among the groups (P<0.001). Proteinuria was detected in both the PE groups, and normotensive pregnant women had negative results for the dipstick test (Table 1).

**Table 1:** Clinical and demographic characteristics of pregnant women enrolled in the study

Parameters	Normotensive pregnant (n = 102)	Early-onset PE (n = 57)	p-value*	Late-onset PE (n = 49)	p-value*
Age (years)	24.97 ± 0.61	26.79 ± 0.95	0.096	26. 52 ± 0.97	0.167
GA (weeks)	32.80 ± 0.44	30.24 ± 0.36	0.001^a^	36.24 ± 0.26	<0.001^a^,^b^
BMI (Kg/m²)	24.27 ± 0.46	24.55 ± 0.74	0.742	24. 45 ± 1.40	0.869
GWG (Kg)	10.51 ± 0.56	12.37 ± 0.94	0.080	15.22 ± 1.09	<0.001^a^
SBP (mmHg)	108.0 ± 0.82	169.6 ± 2.28	<0.001^a^	186.5 ± 16.75	<0.001^a^
DBP (mmHg)	70.20 ± 0.70	108.4 ± 1.79	<0.001^a^	124.0 ± 17.92	<0.001^a^
24-h-Pr (g/24h)	ND	3.44 ± 0.45		3.62 ± 0.68	

GA, gestational age (diagnosis/sample collection); BMI, body mass index; GWG, gestational weight gain; SBP, systolic blood pressure; DBP, diastolic blood pressure; 24-h-Pr, proteinuria; ND, not determined (but, with negative dipstick test); PE, Preeclampsia. Values are the mean ± s.e.m. Significant P values are indicated as follow:

a P < 0.05 vs. normotensive pregnant.

b P < 0.05 vs. early-onset PE

Genotypes and alleles distribution are presented in table 2. The distribution of genotypes for the *DDAH2* polymorphisms showed no deviation from Hardy-Weinberg equilibrium. No significant differences were found for alleles and genotypes frequencies when compared the early-onset or late-onset PE groups with the normotensive pregnant group (P>0.05) (Table 2). The genotype frequencies were also compared considering the allele carriers (Table 2).

**Table 2:** Genotype and allele frequencies for *DDAH2* polymorphisms in normotensive pregnant compared to both early-onset and late-onset PE

Genotype or allele	Normotensive pregnant (n = 204)	Early-onset PE (n = 124)	OR (95% CI)	p-value	Late-onset PE (n = 92)	OR (95% CI)	p-value
rs2272592 (-1415 G/A)							
GG	67 (81%)	43 (77%)	1.000 (Reference)	–	39 (93%)	1.000 (Reference)	–
GA	16 (19%)	13 (23%)	1.266 (0.554-2.892)	0.671	3 (7%)	0.322 (0.088-1.176)	0.112
AA	0 (0%)	0 (0%)	–	–	0 (0%)	–	–
G	150 (90%)	99 (88%)	1.000 (Reference)	–	81 (96%)	1.000 (Reference)	–
A	16 (10%)	13 (12%)	1.231 (0.567-2.672)	0.689	3 (4%)	0.347 (0.098-1.227)	0.127
rs805304 (-1151 C/A)							
CC	34 (34%)	23 (37%)	1.000 (Reference)	–	23 (50%)	1.000 (Reference)	–
CA	44 (45%)	32 (52%)	1.075 (0.534-2.161)	0.860	17 (37%)	0.571 (0.264-1.234)	0.176
AA	21 (21%)	7 (11%)	0.492 (0.180-1.348)	0.228	6 (13%)	0.422 (0.147-1.208)	0.141
CA+AA	65 (66%)	39 (63%)	0.887 (0.457-1.719)	0.737	23 (50%)	0.523 (0.256-1.066)	0.099
C	112 (57%)	78 (63%)	1.000 (Reference)	–	63 (68%)	1.000 (Reference)	–
A	86 (43%)	46 (37%)	0.768 (0.484-1.217)	0.295	29 (32%)	0.599 (0.355-1.010)	0.070
rs805305 (-449 C/G)							
CC	39 (38%)	22 (36%)	1.000 (Reference)	–	22 (49%)	1.000 (Reference)	–
CG	43 (42%)	29 (48%)	1.196 (0.591-2.416)	0.720	13 (29%)	0.535 (0.238-1.206)	0.158
GG	20 (10%)	10 (16%)	0.886 (0.352-2.228)	1.000	10 (22%)	0.886 (0.352-2.228)	1.000
CG+GG	63 (52%)	39 (64%)	1.097 (0.568-2.119)	0.867	23 (51%)	0.647 (0.318-1.314)	0.276
C	121 (59%)	73 (60%)	1.000 (Reference)	–	57 (63%)	1.000 (Reference)	–
G	83 (41%)	49 (40%)	0.978 (0.619-1.546)	1.000	33 (37%)	0.844 (0.505-1.408)	0.604

CI, Confidence Interval; OR, odds ratio; * P < 0.05 vs. normotensive pregnant group

**Figure 1:**
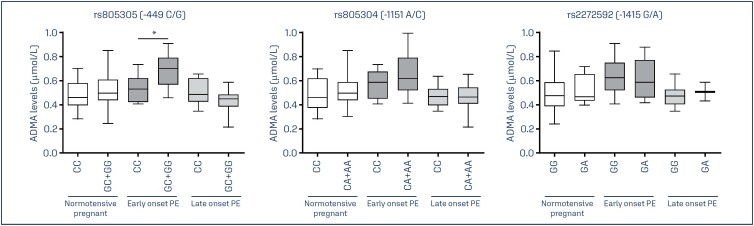
ADMA levels in normotensive pregnant women and pregnant women with early-onset and late-onset PE grouped according to genotypes for the *DDAH2* polymorphisms -449G/C (rs805305), -1151A/C (rs805304), and -1415G/A (rs2272592). The box and whiskers plots show range and quartiles. The boxes extend from the 25th percentile to the 75th percentile, with a line at the median. The whiskers show the highest and the lowest values

We have previously reported ADMA values for 50 normotensive pregnant women and for 54 pregnant with PE,^(13)^ which were increased in early-onset PE [0.66 μmol/L (0.52–0.78)] compared to late-onset PE [0.47 μmol/L (0.42–0.60)] (*P*=0.001) and to the normotensive pregnant group [0.48 μmol/L (0.42–0.58)] (P=0.001).^(13)^ In the present study, we examined the effects of *DDAH2* genotypes on ADMA levels considering the normotensive control group and the pregnant women with early-onset and late-onset PE. We found no effects of *DDAH2* genotypes in the normotensive pregnant group Moreover, we found no effects of genotypes for the -1151C/A and -1415G/A polymorphisms on ADMA levels in pregnant with PE (P>0.05) (Figure 1). However, pregnant women with early-onset PE carrying the GC and GG genotypes for the -449G/C polymorphism showed higher ADMA levels than those carrying the CC genotype (P=0.01) (Figure 1).

The distribution of *DDAH2* haplotypes is shown in table 3 and table 4. Notably, the G-C-G haplotype was more common in late-onset PE than in the normotensive group considering the additive model (P=0.003) (Table 3) and the dominant model (P=0.002) (Table 4). However, no significant difference was found between early-onset PE and normotensive pregnant women in the additive model (P=0.064) (Table 3), or in the dominant model (P=0.018, after Bonferroni correction) (Table 4).

**Table 3:** Frequencies of haplotypes formed by alleles of the *DDAH2* SNPs -1415 G/A, -1151 C/A and -449 C/G in the normotensive pregnant and pregnant with early-onset PE and late-onset PE, considering the additive model

Haplotypes	Normotensive pregnant (N = 204)	Early-onset PE (n = 124)	P-values	OR (95% CI)	Late-onset PE (n = 92)	P-values	OR (95% CI)
G-C-C	0.5347	0.5098	0.893	1.000 (Reference)	0.5574	0.689	1.0000 (Reference)
G-A-G	0.3024	0.2843	0.541	0.9584 (0.5566-1.6502)	0.2055	0.126	0.7200 (0.3984-1.3014)
A-A-G	0.0837	0.0593	0.676	0.7937 (0.2605-2.4180)	0.0358	0.093	0.4267 (0.1159-1.5709)
G-C-G	0.0207	0.0632	0.064	2.7106 (0.7852-9.3563)	0.1274	0.003*	3.2856 (1.1145-9.6864)
G-A-C	0.0417	0.0274	0.500	0.7102 (0.1907-2.6447)	0.0739	0.384	1.4223 (0.4754-4.2547)
	^a^ Global-stat = 5.85076, df = 6, P-val = 0.44011				^b^ Global-stat = 13.232, df = 5, P-val = 0.021297*		

*DDAH2*, Dimethylarginine Dimethylamine Hydrolase 2; CI, Confidence Interval; OR, odds ratio;

* Pc < 0.01 (0.05/total number of possible haplotypes) was considered significant versus normotensive pregnant, to correct for the number of comparisons made (Bonferroni’s correction)

**Table 4:** Frequencies of *DDAH2* haplotypes formed by alleles of -1415 G/A, -1151 C/A and -449 C/G polymorphisms in normotensive pregnant and in early and late-onset preeclampsia, considering the dominant model

Haplotypes	Normotensive Pregnant (n = 102)	Early-onset PE (n = 57)	P-value	Late-onset PE (n = 49)	P-value
G-C-C	0.5347	0.5098	0.700	0.5574	0.803
G-A-G	0.3024	0.2843	0.690	0.0358	0.067
A-A-G	0.0837	0.0593	0.704	0.2055	0.096
G-C-G	0.0207	0.0632	0.018	0.1274	0.002*
G-A-C	0.0417	0.0274	0.604	0.0739	0.244
	^a^ Global-stat = 8.61623, df = 6, P-val = 0.19634			^b^ Global-stat = 15.31869, df = 6, P-val = 0.01792	

*DDAH2,* Dimethylarginine Dimethylamine Hydrolase 2; CI, Confidence Interval; OR, odds ratio;

* Pc < 0.01 (0.05/total number of possible haplotypes) was considered significant versus normotensive pregnant, to correct for the number of comparisons made (Bonferroni’s correction)

## Discussion

The main novel findings reported in the present study were: (1) pregnant with early-onset PE carrying the GC and GG genotypes for the *DDAH2* -449G/C (rs805305) polymorphism showed increased ADMA levels; and (2) the haplotype G-C-G was found to be associated with the risk for late-onset PE.

Deficiency in DDAH2 activity contributes to high ADMA levels, which compromises the NO synthesis.^(20)^ Our group has found increased ADMA plasma levels in early-onset severe PE compared to late-onset severe PE.^(13)^ Since DDAH enzymes are directly involved in the catabolism of ADMA, it would be expected that *DDAH2* polymorphisms could be associated with PE. It is known that approximately 20% of ADMA is excreted in the urine, and that renal dysfunction is often observed in PE. Thus, it could be inferred that part of the plasma ADMA in preeclamptic women can be related to renal dysfunction. Moreover, it was shown that DDAH can be inactivated in the renal tubules by proteinuria, leading to ADMA accumulation.^(21)^ Oxidative stress, increased glucose and homocysteine plasma levels are other factors that may also contribute for the reduction of DDAH activity.^(22)^ Higher oxidative stress in preeclamptic women^(23)^ and higher homocysteine levels have already been described in mild and severe PE when compared to normotensive pregnant women.^(24)^

Although no previous study has examined the effects of *DDAH2* polymorphisms on ADMA levels in PE, the C allele of the -1151C/A polymorphism was associated with the highest ADMA levels in subjects with type 2 diabetes,^(25)^ and in patients with chronic renal failure.^(18)^ Moreover, the G allele and GG genotype of -449C/G polymorphism were found to be associated with reduced DDAH2 activity.^(26,27)^ Conversely, the AA genotype of -1151C/A polymorphism was associated with an increased prevalence of hypertension.^(28)^ In addition, the presence of at least one A allele was related to increased ADMA levels, and it was also associated with the development of cardiovascular disease.^(29)^ However, other study found no relationship between genotypes of *DDAH2* polymorphisms and ADMA levels and endothelium-dependent vasodilation or with flow-mediated dilatation measurement.^(30)^

To our knowledge, no previous study has examined whether *DDAH2* haplotypes were associated with early-onset and late-onset PE. We have shown for the first time that the G-C-G haplotype may affect the risk of late-onset PE. The haplotype is composed by the combination of alleles for the -1415G/A, -1151C/A, and -449C/G polymorphisms, respectively. It is known that haplotypes may offer improved genetic information in association studies compared with the analysis of single genetic markers,^(31)^ and they can capture the combined effects of causal variants.^(32)^ However, these alleles could also be in linkage disequilibrium with other functional polymorphisms in the promoter region of *DDAH2* gene not included in this study. Regarding functional role, the -1151A/C (rs805304) polymorphism is likely to affect transcription factor binding according to its score 2c at RegulomeDB,^(33)^ which suggests that it may affect *DDAH2* transcription. The three *DDAH2* polymorphisms examined are considered expression Quantitative Trait Loci (eQTLs) for *DDAH2* expression in several tissues according to GTEx portal (https://gtexportal.org/),^(34)^ and the alternative alleles for each polymorphism was associated with decreased expression. In agreement with this functional evidence, we found here that ADMA levels were higher in pregnant women with early-onset PE carrying the GC+GG genotype than in carriers of the CC genotype for the rs805305 polymorphism. The GC and GG genotypes are associated with lower *DDAH2* expression, which could contribute to high ADMA levels.

The present has limitations. First, we have studied a relatively small number of preeclamptic women, and ADMA levels were determined in a smaller number of pregnant women. Despite this limitation, we have shown the effect of *DDAH2* genotypes on ADMA levels. Moreover, we found significant associations of *DDAH2* haplotypes with the risk of PE, and it is important to consider that the G-C-G is a common haplotype (>12% frequency in the late-onset PE group), thus highlighting its importance. Second, we have not measured placental or tissue ADMA levels and, therefore, its importance is not known at tissue level. In summary, although genetic polymorphisms may have clinical implications for PE risk, they are not established as the unique factors responsible for the disease, considering the multifactorial character of PE.

## Conclusion

We found that genotypes for the *DDAH2* polymorphisms -1151C/A (rs805304) and -449G/C (rs805305) may affect ADMA levels in pregnant with PE, and the *DDAH2* haplotype G-C-G was found to be associated with the risk for late-onset PE.
